# Contributions of modern Gobi Desert to the Badain Jaran Desert and the Chinese Loess Plateau

**DOI:** 10.1038/s41598-018-37635-y

**Published:** 2019-01-30

**Authors:** Xunming Wang, Diwen Cai, Jimin Sun, Huayu Lu, Wenbin Liu, Mingrui Qiang, Hong Cheng, Huizheng Che, Ting Hua, Caixia Zhang

**Affiliations:** 1 Key Laboratory of Water Cycle and Related Land Surface Processes, Institute of Geographic Sciences and Natural Resources Research, Chinese Academy of Sciences, Beijing, 100101 China; 2 University of Chinese Academy of Sciences, Beijing, 100049 China; 3 Key Laboratory of Cenozoic Geology and Environment, Institute of Geology and Geophysics, Chinese Academy of Sciences, Beijing, 100029 China; 4 School of Oceanographic and Geographic Sciences, Nanjing University, Nanjing, 210023 China; 5 Key Laboratory of Western China’s Environmental Systems (Ministry of Education), College of Earth and Environmental Sciences, Lanzhou University, Lanzhou, 730000 China; 6 State Key Laboratory of Earth Surface Processes and Resource Ecology, Beijing Normal University, Beijing, 100875 China; 7 State Key Laboratory of Severe Weather (LASW), Institute of Atmospheric Composition, Chinese Academy of Meteorological Sciences, Beijing, 100081 China; 8 Key Laboratory of Desert and Desertification, Cold and Arid Regions Environmental and Engineering Research Institute, Chinese Academy of Sciences, Lanzhou, 730000 China

## Abstract

It is well known that the Gobi Desert is the dominant source area of the Badain Jaran Desert (BJD) and the Chinese Loess Plateau (CLP). However, due to the absence of quantitative analyses, there are nearly no exact assessments of its actual contribution. Combinations of field investigations, wind tunnel experiments, and wind field analyses revealed that the potential erosion depth on modern Gobi Desert varied between 0.41 and 0.89 mm a^−1^. Results indicated it would take an average theoretical time of 80.8 ka and 4,475.9 ka to form the current dimensions of the BJD and CLP, respectively, which means the Gobi Desert may provide substantial sand sources to the modern BJD, while its contribution to the loess of modern CLP might be overestimated despite it was the key sources of the CLP in Quaternary.

## Introduction

**Figure 1:**
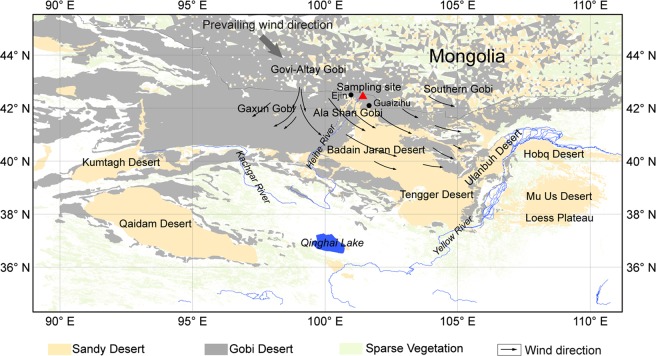
Location of the Gobi Desert, Badain Jaran Desert, Chinese Loess Plateau, and the sampling site. The black dots indicate the meteorological stations employed in this study.

The Gobi Desert, Badain Jaran Desert (BJD), and the Chinese Loess Plateau (CLP, Fig. 1), located in northwestern China and southern Mongolia, are known as the key areas of dust emissions in Central Asia^1^, regions owning the highest sand dunes in the world^2^, and the cradle of Chinese civilization^3^, respectively. The Gobi Desert, developed from the Upper Pleistocene to the Holocene^4,5^ by aeolian-fluvial interactions^6^, and also called as “desert pavement” or “stony desert” being characterized by “wide, shallow basins of which the smooth rocky bottom is filled with sand, silt or clay, pebbles or, more often, with gravel”^7,8^ (Supplementary Information S1), was considered as one of the sand sources of the BJD^9^ and the important loess source of the CLP^10,11^. However, there were still some debates on the source of the BJD and CLP. For instance, some studies^9,12^ believed that the sand sources of the BJD were originated primarily from lacustrine and fluvial processes, the main sources of which are the weathered and denuded products of the underlying Mesozoic and Cenozoic sandstones, sandy conglomerate, and clastic rocks. In addition, the lacustrine sediments^13^ and some alluvial fans^14,15^ developed in the west and northwest of the desert also provided sand sources for the BJD. Based on the studies so far, the potential loess sources of the CLP were mainly originated from the mountains and sandy deserts^16^, Mongolian gobi desert^17^, the Yellow River sediments^18,19^, and the northern Tibetan Plateau^20^.

According to previous studies (Supplementary Information S2), it is clear that under the modern circulations the Gobi Desert is not only the key provider of the sand sources of the BJD but also the potential loess sources of the CLP. However, there is little attention paid to the exact contributions of the Gobi Desert, which is especially important to elucidate the provenance of sand and loess of the BJD and CLP. Therefore, by utilizing the comprehensive field investigations, wind tunnel experiments, simulations, and the remote-sensing analyses, here we quantitatively evaluated the contributions of Gobi Desert on the sand and loess sources of BJD and CLP under the modern wind regimes. The analyzed results showed that under modern wind regime the potential erosion rates on the Gobi Desert varied between 0.41 and 0.89 mm a^−1^, which were potential sand sources for the BJD formation with relatively low contributions to the loess of the CLP.

## Materials and Methods

On the Gobi Desert, the gravels cover most of the surface, leaving only about 10% covered by other landforms such as mobile sand sheets, dunes, wadis, and residual hills. The primordial underlying landforms of gobi deserts are alluvial fans, playas, and wadis, and the dominant sediment sources are the adjacent Gobi Altai Mountains, the Heihe River, and the highlands of southern Mongolia, which are transported by intermittent floods from the upper Pleistocene to the early Holocene^21,22^. 15 intact gobi surface samples were collected for further wind tunnel experiments, and more details of sampling strategies are provided in Supplementary Information S3.

Wind tunnel experiments were performed in the Key Laboratory of Desert and Desertification, Chinese Academy of Sciences, China. Details of the experimental processes are described in Supplementary Information S4. After all wind-tunnel experiments, the collected aeolian materials were weighed and proceeded by particle size analysis, and more details of particle size analyses are described in Supplementary Information S5. Once the particle size analyses were finished, the aeolian transports under different wind speeds could be acquired according to the results of wind tunnel experiments (Supplementary Information S6). Additionally, wind velocity observations during 1951 to 2015 from 2 weather stations (Ejin and Guaizihu, Fig. 1) within the Gobi Desert were employed to further analyses. These data were recorded in accordance with the World Meteorological Organization (WMO) and China National Meteorological Center (CNMC) standards. Because most datasets started after 1960, only the wind records from 1960 to 2015 were used to evaluate the temporal variation in the aeolian transport potentials. More detailed descriptions of the data processing are shown in Supplementary Information S7.

## Results and Discussion

**Figure 2:**
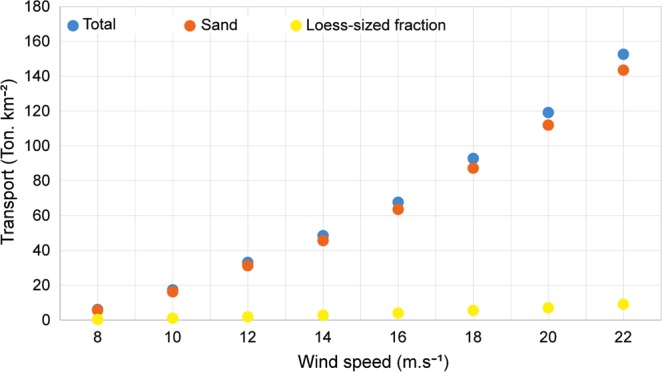
The total sand and dust transports under different wind speed. More details of the transport results are shown in Supplementary Fig. S7.

The wind tunnel experiments and the particle size analysis showed that the averaged sand fractions (50~2000 μm in diameter) and loess-sized fractions (<50 μm in diameter) were 93.9% and 6.1%, respectively. The average total transports of the 12 samples under wind velocity of 8 to 22 m s^−1^ spanned between 6.06 and 152.65 g m^−2^, and when considering the proportions of fine fractions (<50 μm in diameter) in transported materials, the average sand transports under wind velocity of 8 to 22 m s^−1^ varied between 5.66 and 143.59 g m^−2^ (Fig. 2).

From 1960 to 2015, the potential sand transports in Ejin varied between 112 and 2,722 g m^−2^ a^−1^ with an average of 1,047 g m^−2^ a^−1^, while in Guaizihu those figures were 1,471, 3,399, and 2,265 g m^−2^ a^−1^, respectively (Supplementary Fig. S7). When considering the proportions of the fine fractions emitted from the source regions and settled *in situ* again (Supplementary Information S7), and considering the areas of the upwind Gobi which have potential effects on the BJD and the CLP (Supplementary Information S8), the results showed that the average annual sand and loess-sized transports from 1960 to 2015 were 0.16 and 0.34 km^3^ a^−1^, 0.0029 and 0.0062 km^3^ a^−1^ in Ejin and Guaizihu, respectively (Table 1).

**Table 1:** Summary of the annual sand and loess-sized fraction transports availability in the Gobi Desert (km^3^ a^−1^).

Estimation range	Sand transports		Loess-sized fraction transports	
	Ejin Station	Guaizihu Station	Ejin Station	Guaizihu Station
Minimum	0.02	0.22	0.0003	0.0041
Maximum	0.41	0.52	0.0074	0.0092
Average	0.16	0.34	0.0029	0.0062

At present, the total sand dimensions of the BJD were about 1.292 ± 0.362 × 10^4^ km^3^ (Supplementary Information S9), while those for the modern CLP varied from 8,908 to 18,180 km^3^ with an average value of 12,980 km^3^ (Supplementary Information S10). Therefore, assuming that the Gobi Desert is the sole source of the sand and loess, it will at most take 103.4 ka and 6,269.0 ka (i.e., theoretical time) to reach the modern dimensions of the BJD and CLP, respectively (Table 2). Given that the Gobi Desert was developed in the Upper Pleistocene (circa 420 ka B.P.), with the knowledge of the age of BJD (Supplementary Information S11), the experimental and statistical results showed that under the modern wind regime the Gobi Desert could provide nearly all sand sources for the BJD formation. By comparison, fluvial processes, taken Heihe River for instance (Fig. 1), would spend at least 1,681 ka to supply adequate sand materials to achieve the modern dimensions of the BJD based on the evidence that the Heihe River can only transport 40,000 tons of sand per day^23^ from Qilian Mt. to alluvial fan western of the BJD, which is much longer than most of the known ages of the BJD (Supplementary Information S11). In addition, assuming that the Gobi Desert is also the sole source of the loess in CLP, our experimental results show that at least 3.07 Ma is needed for the CLP development. But, in fact, the acknowledged ages of the basal loess-paleosol sequence (L33) in CLP was about 2.6~2.8 Ma^24,25^, and the wind patterns changed during glacial-interglacial cycles with diverse loess sources as well^26,27^, which suggests that the Gobi Desert could not be reckoned as the sole source. Besides, the results of the erosion rates of loess over the past 25 ka in CLP (Supplementary Information S12) and partly deposition of the fine fractions indicate that the Gobi Desert may not be the main source of loess with very low contributions to the CLP under modern wind regime.

**Table 2:** Scales of the theoretical time needed for the BJD and CLP formation with the Gobi Desert as the sole availability.

Wanted time scales	BJD (ka)	CLP (ka)
Minimum	58.1	3,072.1
Maximum	103.4	6,269.0
Average	80.8	4,475.9

The estimation was based on modern wind regime recorded in Ejin Station as this station is more representative than Guaizihu Station which is in front of a mountain pass with funneling effects (Supplementary Fig. S7) and located in the Gobi Desert zone (Supplementary Fig. S9).

## Conclusions

Although some previous studies had acknowledged that in the Quaternary the Gobi Desert was the key source areas of the Badain Jaran Desert (BJD) and the Chinese Loess Plateau (CLP), there are no quantitative estimations for the potential contributions of the Gobi Desert. Results of comprehensive field investigations, wind tunnel experiments, and the modern wind regime analyses showed that the modern wind erosion depth on the Gobi Desert varied between 0.41 and 0.89 mm a^−1^, and the average theoretical time needed to form the current dimensions of the BJD and CLP were respectively 80.8 ka and 4,475.9 ka based on the rates of transported sand and loess-sized fractions. The aeolian processes of the adjacent Gobi Desert may provide substantial sand sources for the formation of BJD, while its contributions to the loess of the CLP were relatively low. However, based on the calculation of potential transport rates, the changes in wind regime, and the formation and development of the Gobi Desert, there might be some differences between the estimated and actual time to form the current dimensions of the BJD and CLP and further researches are expected to fill the gaps with more precise estimation.

## Supplementary information

Contributions of modern Gobi Desert to the Badain Jaran Desert and the Chinese Loess Plateau
