# Supplementary material for: Contributions of modern Gobi Desert to the Badain Jaran Desert and the Chinese Loess Plateau
Source: Sci Rep. 2019 Jan 30;9:985. doi: 10.1038/s41598-018-37635-y (PMC6353923; doi:10.1038/s41598-018-37635-y)
Supplement: Supplementary file 1 — Contributions of modern Gobi Desert to the Badain Jaran Desert and the Chinese Loess Plateau [file 41598_2018_37635_MOESM1_ESM.docx]

**Contributions of modern Gobi Desert to the Badain Jaran Desert and the Chinese Loess Plateau**

**Xunming Wang^1, 2*^,** **Diwen Cai^1, 2^, Jimin Sun^2, 3^, Huayu Lu^4^, Wenbin Liu^1^, Mingrui Qiang^5^, Hong Cheng^6^,** **Huizheng Che^7^, Ting Hua^8^, Caixia Zhang^8^**

*^1^Key Laboratory of Water Cycle and Related Land Surface Processes, Institute of Geographic Sciences and Natural Resources Research, Chinese Academy of Sciences, Beijing 100101, China*

*^2^University of Chinese Academy of Sciences, Beijing 100049, China*

*^3^Key Laboratory of Cenozoic Geology and Environment, Institute of Geology and Geophysics, Chinese Academy of Sciences, Beijing 100029, China*

*^4^School of Oceanographic and Geographic Sciences, Nanjing University, Nanjing 210023, China*

*^5^Key Laboratory of Western China’s Environmental Systems (Ministry of Education), College of Earth and Environmental Sciences, Lanzhou University, Lanzhou 730000, China*

*^6^State Key Laboratory of Earth Surface Processes and Resource Ecology, Beijing Normal University, Beijing 100875, China*

*^7^State Key Laboratory of Severe Weather (LASW), Institute of Atmospheric Composition, Chinese Academy of Meteorological Sciences, Beijing 100081, China*

*^8^Key Laboratory of Desert and Desertification, Cold and Arid Regions Environmental and Engineering Research Institute, Chinese Academy of Sciences, Lanzhou 730000, China*

**Corresponding author: E-mail: xunming@igsnrr.ac.cn*

**Supplementary Information**

# S1 Gobi Desert and its regional environments

The Gobi Desert is located in northwestern China and southern Mongolia (Figs 1 and S1), including the southern Mongolian Gobi, Ala Shan Gobi, and some parts of the Gaxun Gobi. The Gobi Desert is a hyper arid area in which the annual precipitation seldom exceeds 100 mm with annual evaporation frequently greater than 3,000 mm. The mean annual temperature is ~ 9°C and the mean annual wind velocity is 3.3 m s^-1^. The Gobi desert (also called as "desert pavement" or "stony desert") was described as "wide, shallow basins of which the smooth rocky bottom is filled with sand, silt or clay, pebbles or, more often, with gravel" ^1, 2^ (Fig. S2). In the Gobi Desert, gravels cover most of the surface, leaving only about 10% of the surface covered by other landforms such as mobile sand sheets, dunes, wadis, and residual hills ^3^.


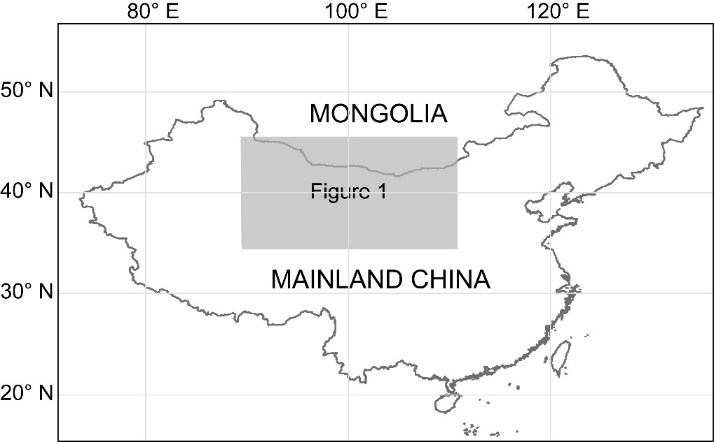


**Figure S1. Location map of the Gobi Desert, Badain Jaran Desert, and the Chinese Loess Plateau.**

**
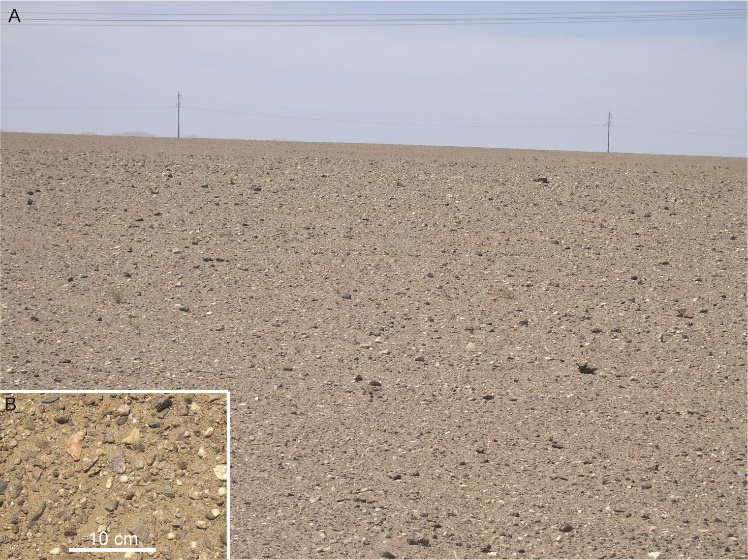
**

**Figure S2. Landscape of Gobi Desert. The location is around 41°52′N, 100°59′E.**

The dominant sediment sources of the Gobi Desert are the adjacent Gobi Altay Mountains, the Heihe River, and the highlands of southern Mongolia. Due to a combination of neotectonic processes and climate change from the upper Pleistocene to the early Holocene ^4, 5^, sediments were carried into the gobi areas by intermittent floods and consequently, there are four combination types for the gobi surfaces and the underlying strata (Fig. S3).

**
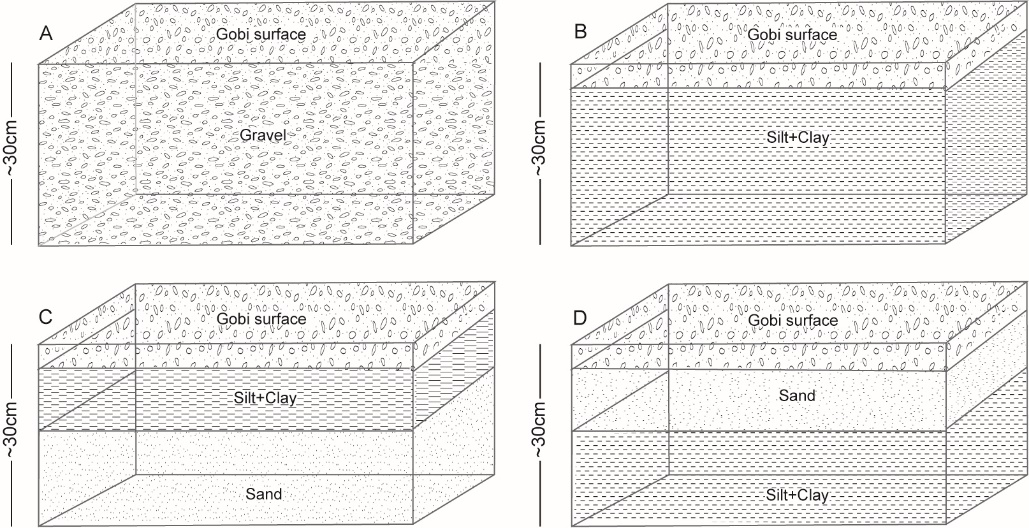
**

**Figure S3. Combinations types for the gobi surfaces and the underlying strata in the Gobi Desert.**

# S2. Possible mechanisms for the potential sources of sand transport and dust emissions on the Gobi Desert

When the gobi desert was developed, it provided abundant materials for the sandy desert formation and the dust emissions in the downwind region^4, 6, 7^. At present, there are several mechanisms proposed for the productions of loose particles on the Gobi surfaces, including glacial grinding, rainfall erosion, aeolian abrasion, snowmelt, and freeze-thaw weathering (Fig. S4). In the Gobi Desert, the first potential source of dust is the underlying fine fractions which are carried into the gobi by ephemeral streams^4^. The second source of erodible fine particles originates from aeolian abrasion, salt and frost weathering, and other physical weathering processes that act on the coarse surface conglomerates^8-12^, and this and other forms of chemical weathering may be the dominant factors responsible for creating the fine fractions in situ^13^.

**
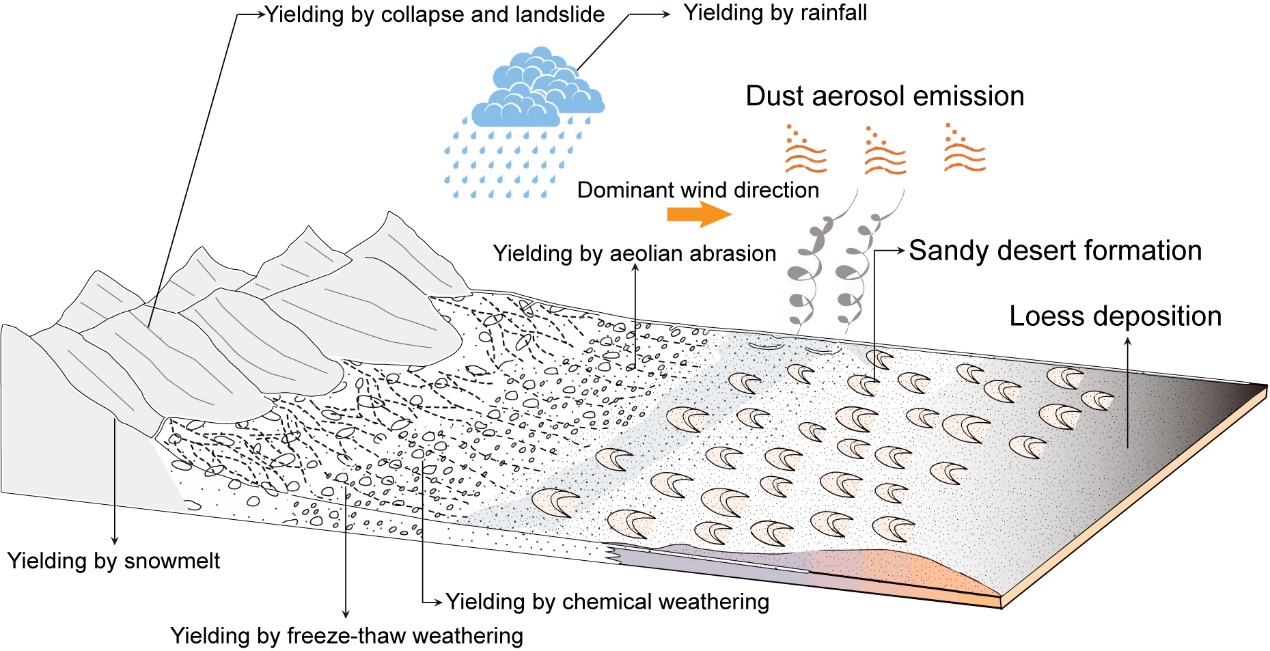
**

**Figure S4. A conceptual model showing the production mechanisms of fine particle size fractions, which are the potential materials for dust emissions and dune system development under aeolian processes.** This figure was drawn by Adobe Illustrator CC 2015 (v19.2.1, https://www.adobe.com/cn/).

# S3 Surface sample collection

15 undisturbed samples of gobi surfaces (Fig. 1), which were representative of the gobi surfaces, were collected by using 120×30×30 cm sample boxes. We sampled surfaces at each site that lacked vegetation cover and that had no biological crusts, that had a smooth and intact surface (sealed, with no cracks), and that showed no signs of anthropogenic impacts. At each site, we selected samples with a range of coverage of the surface by gravel, which were 100 to 200 m apart of each other.

To extract undisturbed samples of the selected gobi surfaces, we placed the sample box on the surface, removed the soil around the box, and then excavated downward for ca. 80 cm without disturbing the sampling faces until the box could be pressed downwards to enclose the surface materials (Fig. S5). A rigid wood sheet was then inserted horizontally to serve as the bottom of the box. The box was covered to protect the surface of the sample, and steel wires were used to wrap the box and ensure that its contents would not change during transport. Unfortunately, three samples were broken during transport, so we only acquired 12 intact samples for use in our wind tunnel experiments and other analyses.


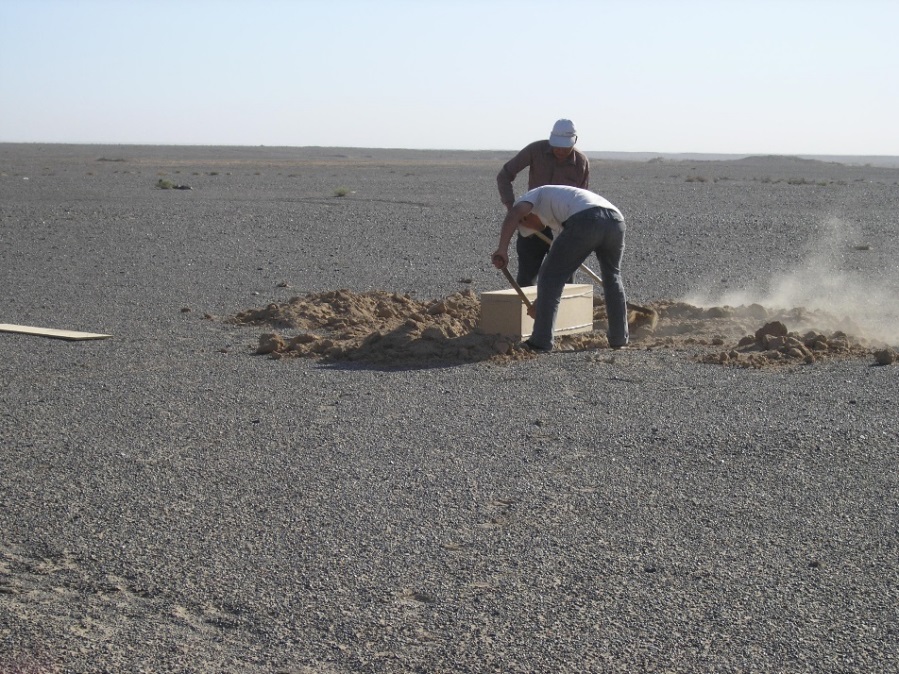


**Figure S5. Sampling in filed. The location is around 41°47′N, 100°59′E.**

# S4 Wind tunnel experiments

The wind-tunnel experiments were performed at the Key Laboratory of Desert and Desertification of the Cold and Arid Regions Environmental and Engineering Research Institute, Chinese Academy of Sciences. The structure of the wind tunnel and its airflow characteristics can be referred in ref. 14.

According to previous studies (e.g., Refs. 15-17), the threshold wind velocity in Gobi Desert is 7 m s^-1^, we started the experiments with a free-stream wind velocity of 8 m s^-1^, and then progressively increased at increments of 2 m s^-1^ and the maximum wind velocity was 22 m s^-1^. For wind velocity, we used a wind profile sampler to measure the wind velocity at heights of 0.3, 0.6, 1.2, 2.4, 4.0, 8.0, 12.0, 16.0, 20.0, and 25.0 cm above the floor of the wind tunnel (Fig. S6b).

The gobi surface sample was positioned in the testing section of the wind tunnel with the surface at the same level as the bottom of the wind tunnel (Fig. S6). At a distance of 30 cm downwind from the sample, a sand trap with 30-cm-width and 30-cm-height was used to collect windblown materials. Because there was no replenishment of surface materials after each run, we used clean wind to acquire the sediment transport rates; as a result, sediment transport decreased after each run for a given sample as a result of progressive depletion of the fine materials. After sediment transport stopped at each wind velocity, we emptied the sediment sampler and weighed the total amount of sediment collected.

Particles larger than 2 mm in diameter on gobi surfaces play an important role in the aeolian transports^18^, so the gravel cover in each sample at the start of each run was photographed by a digital camera. Using Adobe Photoshop V8.0 (Adobe Systems, San Jose, CA), total 48 experimental results with different gravel coverage (Table S1), which included the surface gravel coverage of the modern Gobi Desert, were acquired.


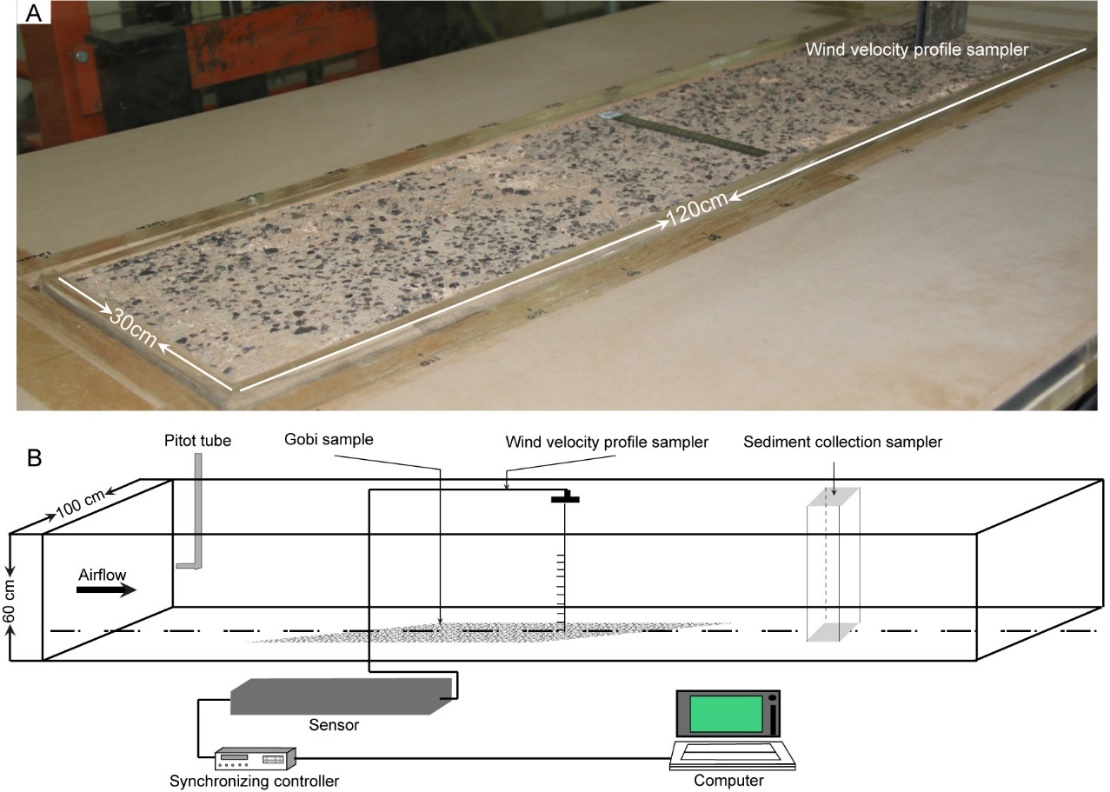


**Figure S6. Schematic diagram of (A) the wind tunnel and sample arrangement used during the wind tunnel experiment, and (B) the surface samples.**

**Table S1. Descriptions of the gravel coverage (%) of the samples**

| Max. | Min. | Mean | Std. |
| --- | --- | --- | --- |
| 42.94 | 5.74 | 23.53 | 8.99 |

# S5 Particle size analyses

After the wind tunnel experiments, the transported sediments collected by the sediment trap were weighed using a high-precision balance. A Mastersizer 2000 (Malvern Co. Ltd., Malvern, UK; sample range between 0.02 and 2000 μm in diameter) was used to measure the particle size distribution of the transported sediments. Comparisons of the results between this method and other methods, such as sieving, hydrometry, and pipetting, were provided by Eshel^19^. Before conducting the particle size measurements, we immersed the sediments in 10% H_2_O_2_ followed by immersion in 12.7% HCl to remove any plant debris and disperse aggregates within the sediments. The sample residue was finally treated with 10 mL of 0.05 M (NaPO_3_)_6_ on an ultrasonic vibrator for 10 min to facilitate dispersion before measuring the particle size.

# S6 Wind tunnel data processing

The wind velocity profile (*u* = *a* ln(*z*) + *b*, where *u* is the wind velocity (m s^-1^), *z* is the height (cm), and *a* and *b* are the fitting coefficients) is determine by fitting the variations of wind velocity with height from above wind tunnel experimental data. According to the method of *u_*_* = *a K* (where *K* is the von Kármán constant of 0.4) to determine shear velocity^20, 21^, the corresponding shear velocities for the axis wind velocities of 8, 10, 12, 14, 16, 18, 20, and 22 m s^-1^ in the wind tunnel testing section were 0.34, 0.40, 0.48, 0.53, 0.57, 0.62, 0.68 and 0.73 m s^-1^. Although the wind velocity of 22 m s^-1^ in the wind tunnel did not achieve the maximum observation value of meteorological stations in gobi desert from 1960 to 2015, the cumulative time for the wind velocity exceeding 22 m s^-1^ was very short. Hence, the experimental wind velocity includes most of the change ranges for the observations of meteorological stations. In addition, existing studies demonstrated that aeolian transport rates from wind tunnel are consistent with the field observations under the same wind velocity^22, 23^. Therefore, our results basically reflected the field actual conditions.

Due to the limitations of the erodible particle productions on Gobi Desert surfaces, it is impossible for the aeolian transports to take place regularly even if the wind velocity is far beyond the threshold. On the other hand, stronger winds will transport not only heavier materials but also materials that would be transported at lower velocities. Therefore, the raw data acquired during the wind tunnel experiments and during the measuring procedures must be re-processed using statistical methods. Here, we used the weighted average method:

$R_{w}=(R_{1}M_{1}+R_{2}M_{2}+\cdots+R_{m}M_{m})/(M_{1}+M_{2}+\cdots+M_{m})$ (1)

where *R*_w_ is the weighted mean of different particle sizes, *R*_1_, …, *R_m_* are the percent of different particle sizes for our experimental wind velocities; *M*_1_, …, *M_m_* are the corresponding contents of the raw transported material.

In general, this particle size of sand grains on Gobi desert surface maybe less than that of natural desert sands^24^. Existing studies demonstrated that the saturated lengths increases with particle size of sand grains ^25-28^, which indicated that the saturated lengths on Gobi desert surface may be less than that on mobile desert surface. However, the gravel cover on Gobi desert surface may reduce the probability of saltating sand grains with particles in bed surface, suggesting that blown sand motion on Gobi desert surface was obviously different from that on mobile desert surface so that the saturated lengths on Gobi desert surface was larger than that on mobile desert surface^15, 17^. In fact, the experimental bed length was shorter than most of the saturated lengths in existing studies. For example, the saturated length was 0.97-2.46 m^25-28^, or equals to only 4-7 m^22^ or over 15 m^29-32^. This means that the experimental aeolian sand motion in this paper is still unsaturated one. For saturated aeolian sand flow, the model of Lettau^33^ was generally used to calculate the flux, which were formulated as follows:

$q=C\frac{\rho_{a}}{g}\sqrt{(\frac{d}{D})}u_{*}(u_{*}^{2}-u_{*t}^{2})$ (2)

where *ρ_a_*, *g*, *d*, *u_*_*, and *u_*t_* are the air density, gravitational acceleration, particle size, friction velocity, and threshold friction velocity, respectively. The two constants are *C* = 6.7 and *D* = 250 µm. The value of *u_*t_* can be calculated using Bagnold’s expression:

$u_{*t}=A\sqrt{\frac{\rho_{s}}{\rho_{a}}gd}$ (3)

where *ρ_s_* is the particle density, and *A* = 0.1.

In general, the flux (*q*) of aeolian sand flow is the product of the incident flux (*Φ*) of aeolian sand flow and the saltation length (*L*) ^25, 34^. For a saturated steady aeolian sand flow, the value of *Φ* is consistent with the emission flux (*ϕ*) from the sand surface. Therefore, *ϕ* can also be expressed as:

 (4)

where *L* is equal to 8 μ_*_^2^/g ^35^.

The emission flux (*ϕ*) was calculated using Equation (4) according to our particle size and friction velocity (Table S2). The experimental aeolian sand transport rates were less than the calculated results in the corresponding condition, which indicated in advance that our experimental aeolian sand flux has not yet achieved the saturated steady. Hence, the data processing is satisfactory.

**Table S2. Calculated theoretical results of the emission flux from a sand surface for different particle sizes with different friction velocities**

| Particle size value (*d, µm*) | Emission flux of aeolian sand flow from mobile sand surface (kg m^-2^ s^-1^) | | | | | | | |
| --- | --- | --- | --- | --- | --- | --- | --- | --- |
|  | Friction velocity (m s^-1^) | | | | | | | |
|  | 0.34 | 0.4 | 0.48 | 0.53 | 0.57 | 0.62 | 0.68 | 0.73 |
| 100 | 0.056517 | 0.074861 | 0.099248 | 0.112416 | 0.125680 | 0.140219 | 0.128340 | 0.171639 |
| 150 | 0.056517 | 0.078264 | 0.108700 | 0.125984 | 0.140761 | 0.158631 | 0.144824 | 0.199224 |
| 200 | 0.056517 | 0.081667 | 0.113426 | 0.135675 | 0.150816 | 0.172795 | 0.155421 | 0.217614 |
| 250 | 0.051807 | 0.078264 | 0.115789 | 0.139551 | 0.157519 | 0.181293 | 0.162486 | 0.232939 |
| 300 | 0.047097 | 0.074861 | 0.115789 | 0.141490 | 0.162546 | 0.186958 | 0.167195 | 0.243156 |
| 350 | 0.037678 | 0.071458 | 0.113426 | 0.141490 | 0.164221 | 0.191207 | 0.169550 | 0.252351 |
| 400 | 0.028258 | 0.064653 | 0.111063 | 0.141490 | 0.164221 | 0.194040 | 0.169550 | 0.258481 |

# S7 Wind regime of Gobi Desert and its impacts on Badain Jaran Desert and Chinese Loess Plateau formation

We employed the wind velocity data from two meteorological stations (Ejin and Guaizihu) to analyze wind regime and temporal aeolian transport rates. The wind data were counted into groups of 7~9, 9~11, 11~13, 13~15, 15~17, 17~19, and >20 m s^-1^, and the corresponding wind velocities in wind tunnel experiments were 8, 10, 12, 14, 16, 18, 20, and 22 m s^-1^. Because existing studies showed that there were no significant differences between wind tunnel experimental results and field observation results^22, 23^, we calculated the total sand and dust transport rates under different wind velocities based on the wind tunnel experimental data. Ejin locates more adjacent to our sample sites for wind tunnel experiments than that of Guaizihu (Fig. 1). In addition, although previous studies suggested^3, 16, 36^ great spatial differences for the wind regimes in the Gobi Desert regions, the global warming has resulted in significant decreases in the wind regime in most regions of the arid Asia^37^ over the past decades, which were not presented in the wind records of Guaizihu. Therefore, here we only use the temporal trends of wind regime in Ejin for further aeolian transport analysis.

In addition, previous studies suggested that once the fine fractions are emitted from the Gobi Desert (e.g., Ref. 38), approximately 30 to 80% of them is settled in situ regions. Chen^39^ estimated that approximately 23% of the emitted fractions are deposited in situ in the Gobi Desert. Therefore, following Chen^39^ we also considered that after the fine material emissions from the Gobi Desert, ~23% of them are emitted outside the Gobi Desert and as the sources of the Chinese Loess Plateau in the region. Combing the wind data, wind tunnel experiments, and the results of the particle size distribution, the sand and dust transports from 1960 to 2015 are shown in Fig. S7.


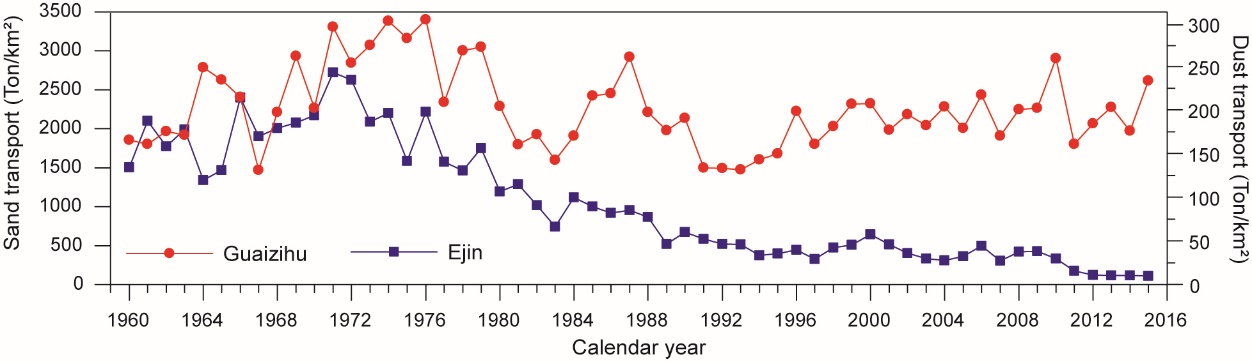


**Figure S7. The annually total sand transports of the two stations from 1960 to 2015 based on 12 gobi samples. More details of the data processing are shown in S6.**

# S8 Areas of the Gobi Desert contributing on Badain Jaran Desert and Chinese Loess Plateau formation

Before determining the areas of the Gobi Desert on Badain Jaran Desert and Chinese Loess Plateau formation, the wind fields in the region are required. Wind velocity dataset as the composite of the zonal and meridional wind data from the monthly ECMWF (European Center for Medium range Weather Forecasts, <https://www.ecmwf.int/en/forecasts/datasets/reanalysis-datasets/era-interim>) ERA-Interim reanalysis were employed. In addition, the DEM data with a 90 m resolution was acquired from Geospatial Data Cloud (<http://www.gscloud.cn/>); and the data of sandy and gobi deserts are from the Cold and Arid Regions Science Data Centre (<http://westdc.westgis.ac.cn/>) and the Global Land Cover by National Mapping Organizations (GLCNMO, https://globalmaps.github.io/glcnmo.html), which are provided by the Geospatial Information Authority of Japan, Chiba University and collaborating organizations. In addition, the aeolian processes on Gobi Desert mainly occurs in spring (March to May)^36^, and therefore, we employed the wind fields during spring for further analyses, and the wind fields are shown in Figure S8.


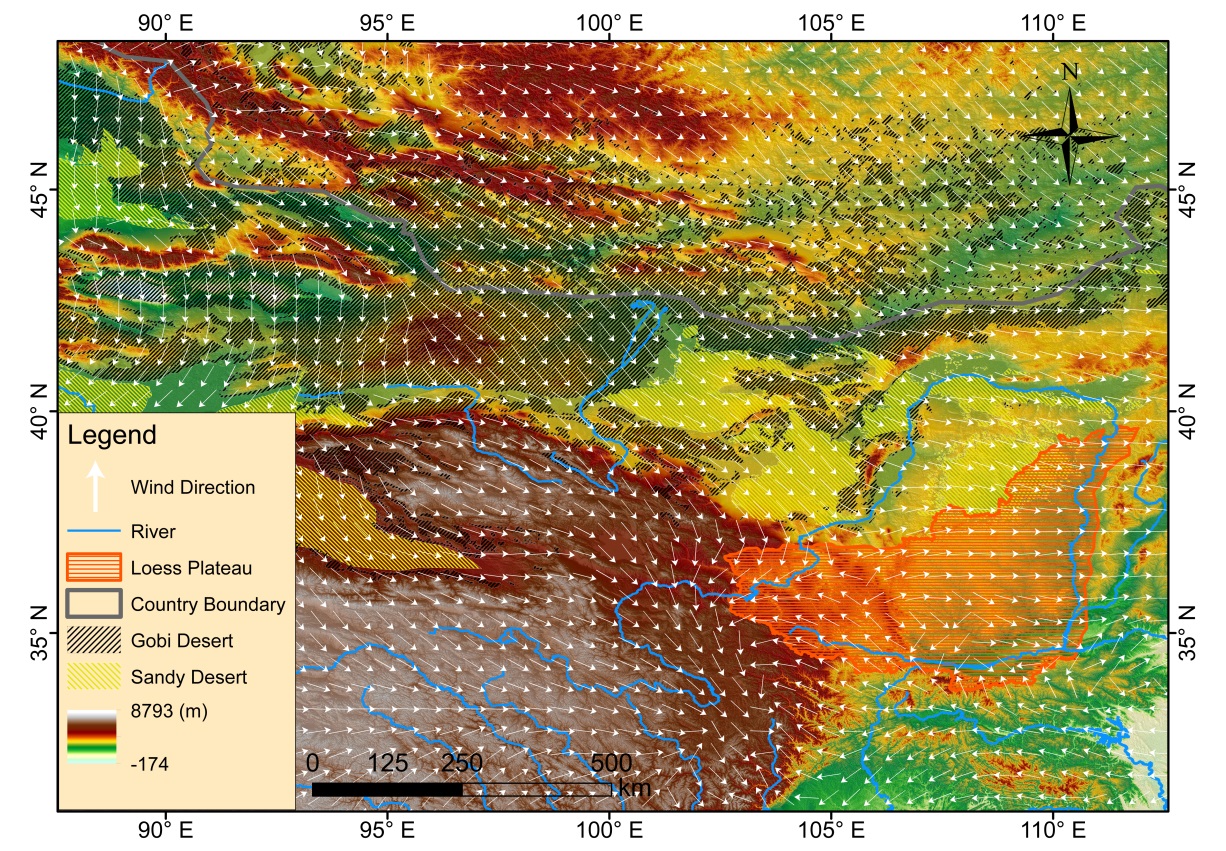


**Figure S8. Wind field in Spring (March to May) on Gobi Desert.**

Following the wind fields and with the calculation functions of the ArcGIS (V10.2), the areas of the Gobi Desert affecting Badain Jaran Desert and Chinese Loess Plateau formation can be determined. The results show that there are 28.83×10^4^ km^2^ of Gobi Desert that may have potential impacts on Badain Jaran Desert and Chinese Loess Plateau formation (Fig. S9).


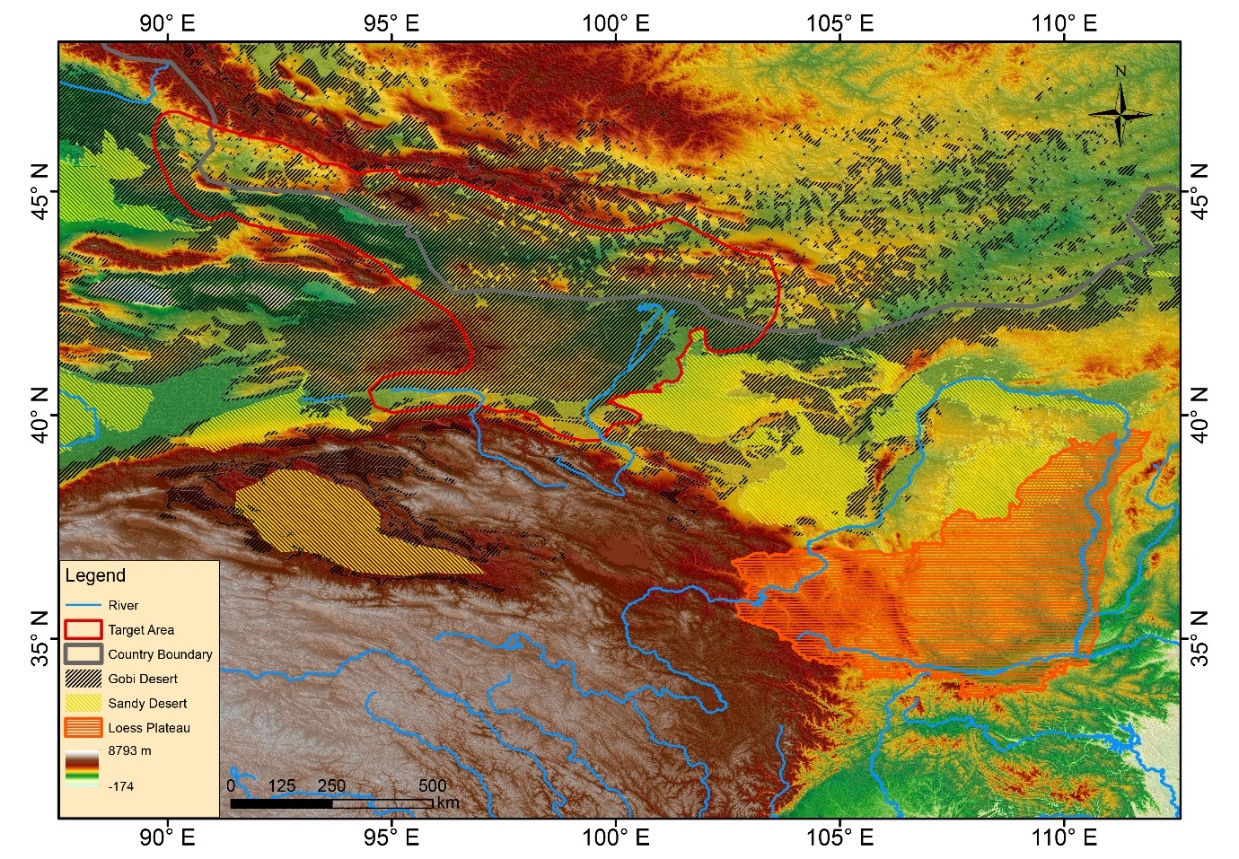


**Figure S9. Area of the Gobi Desert (circled by red lines) that may potentially affect the Badain Jaran Desert and Chinese Loess Plateau formation.**

# S9 Sand volume estimation in the Badain Jaran Desert

Location of the Badain Jaran Desert is shown in Fig. 1. Due to different classifications of the sandy desert, there are still some disputes on the areas of Badain Jaran Desert. For instance, at present the minimum and the maximum estimated area of Badain Jaran Desert is 4.43×10^4^ km^2^ ^40^ and 5.22×10^4^ km^2^ ^41^, respectively; while the extensively acknowledged area is 4.92×10^4^ km^2^ ^42^.

Following Wang et al. (Ref. 43), the maximum depth of the sand layer in Badain Jaran Desert is 356.5 m, and the minimum depth is 73.1 m with an average of 212.6 m (Fig. S10). Therefore, the estimations of areas and depth of sand layer allow us to deduce the maximum, the minimum, and the mean sand volume as 1.859×10^4^, 0.324×10^4^, and 1.046×10^4^ km^3^, respectively. The underground sand volume is 1.039±0.362×10^4^ km^3^ (Table S3).


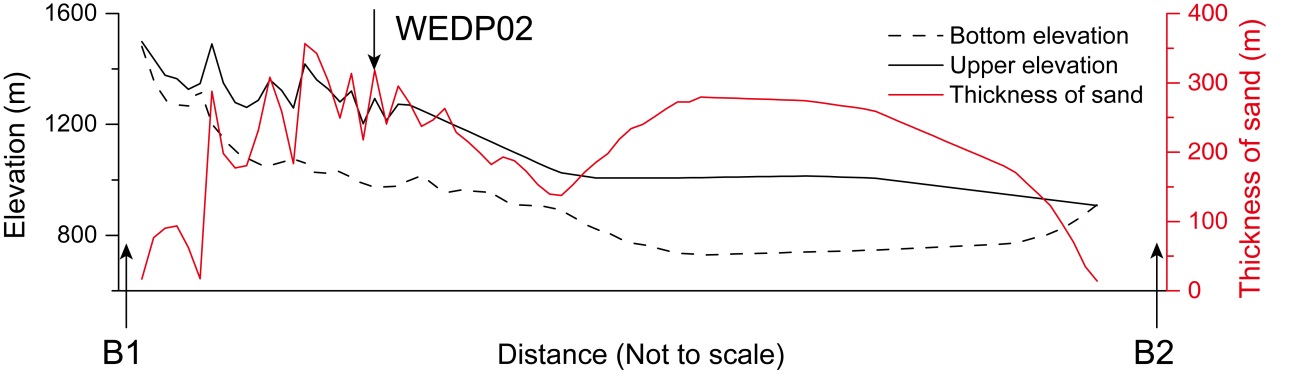


**Figure S10. Section of the underground sand layer depth in the Badain Jaran Desert.**

**Table S3. Estimations of the underground sand volume in Badain Jaran Desert**

| Underground sand volume  (×10^4^ km^3^) | Area × Thickness | A_max_ | A_min_ | A_mean_ |
| --- | --- | --- | --- | --- |
|  | T_max_ | **1.859** | 1.579 | 1.754 |
|  | T_min_ | 0.381 | **0.324** | 0.360 |
|  | T_mean_ | 1.109 | 0.942 | **1.046** |
| Average volume (×10^4^ km^3^) | **1.039±0.362** | | | |

The aboveground sand volume in Badain Jaran Desert was estimated by DEM (Fig. S11), which comes from Geospatial Data Cloud (GDEMDEM, 30 m resolution, <http://www.gscloud.cn/>). The boundary data of the Badain Jaran Desert come from the Cold and Arid Regions Science Data Centre, China (<http://westdc.westgis.ac.cn/>), and the area of the Desert is 4.92×10^4^ km^2^ as previous studies suggested.


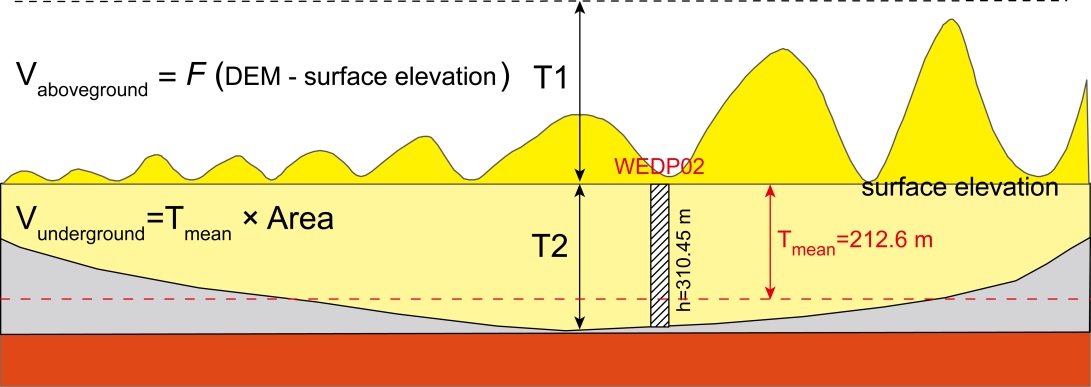


**Figure S11. Schematic diagram of sand volume calculations in the Badain Jaran Desert.**

Throughout the Badain Jaran Desert, 121 controlling sites were arranged at about 30 km interval (Fig. S12). When the layout was finished, these sites were resampled with 30 m resolution by Kriging method, and consequently, the aboveground sand volume was acquired, which was 0.253±0.0004×10^4^ km^3^. Following the above calculation results, the total sand volume of the Badain Jaran Desert is 1.292±0.362×10^4^ km^3^ with the maximum and minimum volume of 2.130×10^4^ km^3^ and 0.555×10^4^ km^3^, respectively (Table S4).


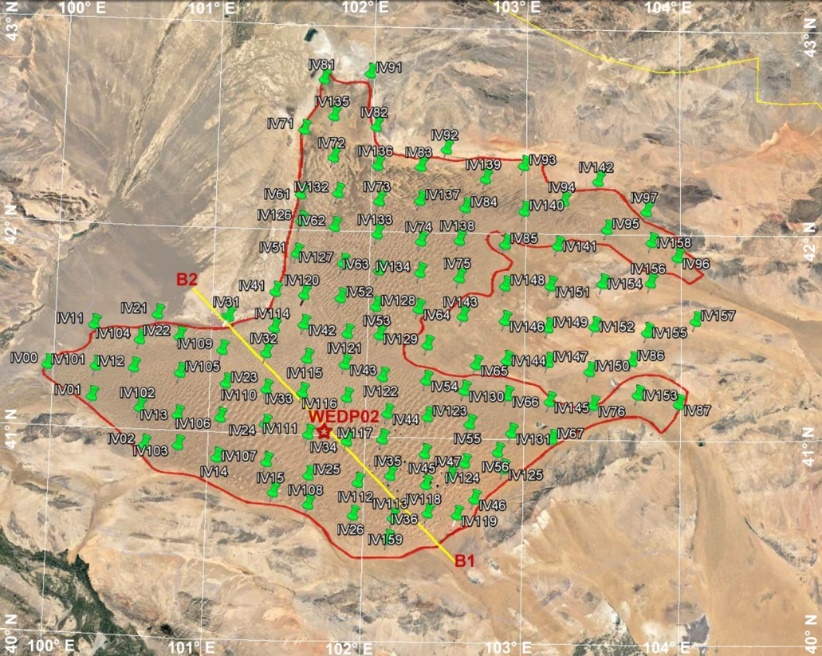


**Figure S12. Layout of the controlling sites in calculating the aboveground sand volume in the Badain Jaran Desert.** The satellite imagery was obtained from Google Earth (v7.1.8.3036, <https://www.google.com/earth/>); the imagery data was Landsat which supported by NOAA US. Navy NGA. GEBCO (the Landsat data source is: NASA Landsat Program, 2003, Landsat ETM+ sceneL71008058_05820031026, SLC-Off, USGS, Sioux Falls, 10/26/2003); and the coordinate grid was created by Adobe Photoshop CC 2018 (v19.0, https://www.adobe.com/cn/).

**Table S4. Total sand volume in the Badain Jaran Desert**

| Categories | Desert Area(×10^4^ km^2^) | | | Sand Thickness(m) | | | | Sand Volume(×10^4^ km^3^) | | | |
| --- | --- | --- | --- | --- | --- | --- | --- | --- | --- | --- | --- |
|  | Max | Min | Mean | Max | Min | | Mean | Max | Min | Mean | Average |
| Underground | 5.2162 | 4.43 | 4.92 | 356.5 | 73.1 | | 212.6 | 1.859 | 0.324 | 1.046 | **1.039±0.362** |
| Aboveground | 5.2162 | 4.43 | 4.92 | - | | | | 0.271 | 0.231 | 0.256 | **0.253±0.0004** |
| Sum | - | | | - | | | | 2.130 | 0.555 | 1.302 | **1.292±0.362** |
| Bulk density | **1.9**×**10^3^ kg m^-3^** | | | | | | | | | | |
| Total | **1.292±0.362**×**10^4^ km^3^** | | | | | **2.455±0.688**×**10^16^ kg** | | | | |  |

# S10 Estimations on the loess deposition volume of the Chinese Loess Plateau

There are different definitions of the Chinese Loess Plateau. Traditionally, the broad range of Chinese Loess Plateau has an area of 62.38×10^4^ km^2^,including regions of western Taihang Mountains, east of the Riyue Mountains, east of the Qinghai-Tibet Plateau, north of Qinling Mountains, and south of the Yinshan Mountains. Some studies refers the scope of the Chinese Loess Plateau (MA in Table S8) to the area of the western of Taihang Mountains, east of Riyue Mountains, south of the Great Wall, and north of the Qinling Mountains, with an area of 47.8×10^4^ km^2^. Whereas, other scientists believes that the Chinese Loess Plateau (NA in Table S8) encompasses regions of south of the Great Wall, north of the Qinling Mountains, west of Lvliang Mountains and east of the Qinghai-Tibet Plateau with an area of 26.91×10^4^ km^2^ ^44^ (Fig. S13).


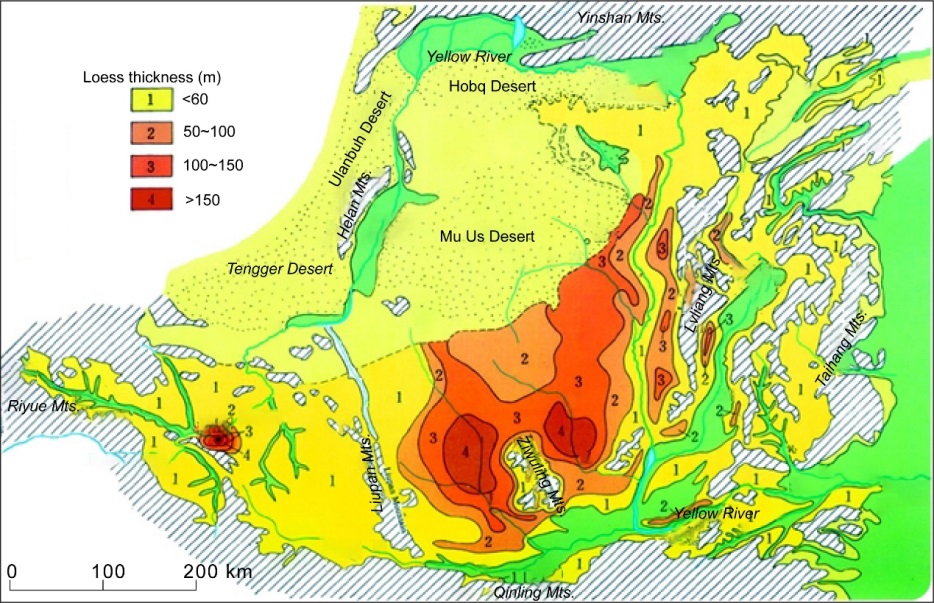


**Figure S13. Location map of the Chinese Loess Plateau (After ref. 45).**

Due to the high soil erosion intensities, the landscapes of Chinese Loess Plateau are discontinuous and there are high differences for the loess thickness in different regions with the maximum reaching up to 400 m and the minimum of several meters ^46, 47^. More details of the loess thickness in different regions are show in Table S5 and Fig. S14.

**Table S5. Loess thickness in Chinese Loess Plateau (After ref. 47).**

| Region | Subarea | Location of typical profile | Location | | Loess-paleosols Profile (m) |
| --- | --- | --- | --- | --- | --- |
|  |  |  | Lon.(°E) | Lat.(°N) |  |
| Western Longxi (West of Liupan Mountains) | Northern part | Dayingliang in Dingxi City | 105.41 | 35.39 | 75.0 |
|  |  | Huajialing in Tongwei County | 105.02 | 35.38 | 1.5 |
|  |  | Shuiquan Dashuiying in Jingyuan County | 104.51 | 37.21 | ~4.0 |
|  |  | Baicaoyuan in Huining County | 104.99 | 36.22 | 280.0 |
|  |  | Ruoli in Jingyuan County | 104.60 | 36.36 | ~150.0 |
|  |  | Jiuzhoutai and Gaolan Mountain in Lanzhou | 103.79 | 36.10 | 287.0 |
|  |  | Xijin Village in Lanzhou | 103.76 | 36.03 | 409.9 |
|  |  | Huanghe River terrace in Minhe County | 102.93 | 36.31 | 100.0 – 150.0 |
|  | Southern part | Wushan County and Tianshui City | 105.45 | 34.61 | ~6.0(<50.0) |
| Eastern Longxi (East of Liupan Mountains) | Southern part | Haodian at southern Yongshou Mountain | 108.08 | 34.83 | ~30.0 |
|  |  | Chishui River at southern Yongshou Mountain | 108.03 | 34.78 | ~18.0 |
|  | Northern part | Dazhaizi Village and Laozhuang Village in Pingliang City | 107.76 | 35.08 | 163.5 |
|  |  | Huoxianggou in Xifeng, Qingyang City | 107.49 | 35.45 | 177.0 |
|  |  | Taiyu Village at northern Yongshou Mountain | 108.05 | 34.93 | 152.5 |
|  |  | Jiantou Village in Pingliang City | 106.72 | 35.58 | 175.0 |
|  |  | Anjia Village in Pingliang City | 107.00 | 35.49 | 137.0 |
|  |  | Yegou Village in Jingchuan County | 107.30 | 35.23 | 128.0 |
|  |  | Tuoshuigou Village in Changwu County | 107.83 | 35.16 | 160.0 |
|  |  | Xiaodong Village in Changwu County | 107.95 | 35.35 | 138.0 |
|  |  | Dafosi in Bin County | 108.00 | 35.07 | 155.0 |
|  |  | Yejihong in Xunyi County | 108.31 | 35.16 | 156.0 |
|  |  | Kaibian Village in Zhenyuan County | 107.05 | 35.79 | 175.0 |
|  |  | Jiaojiaqu Village in in Zhenyuan County | 107.27 | 35.77 | 175.0 |
|  |  | Litian Village in Ning County | 107.88 | 35.49 | 143.0 |
|  |  | Xigou in Zhengning County | 108.35 | 35.38 | 140.0 |
| The Southern region of Ningxia Hui Autonomous Region | Intermountain basin | Southern Xiang Mountain | 105.09 | 37.08 | 2.0 – 5.0 |
|  |  | Nanshanmen Village in Haiyuan County | 105.62 | 36.65 | 11.2 |
|  | Valley terrace | Qingshui River at Guyuan County | 106.18 | 36.30 | 20.0 |
|  |  | Shuidonggou in Lingwu County | 106.52 | 38.30 | ~8.0 |
|  | Region of watershed | Qinqigou Shibeiwan at Guyuan County | 106.67 | 36.19 | 35.0 |
|  |  | Heima fountain at Guyuan County | 106.43 | 36.18 | ~40.0 |
| Northern Shaanxi Province | Northern part | Tufosi at Wuqi County | 108.27 | 36.81 | 195.8 |
|  |  | Laoguajie at Ansai County (Yanhe River vallay) | 109.23 | 37.16 | 127.1 |
|  |  | Majiagou at Yanchuan County (Qingjian River vallay) | 110.27 | 36.81 | 193.3 |
|  | Southern part | Heimugou at Luochuan County | 109.44 | 35.72 | 144.7 |
|  |  | Beilongting at Hancheng City | 110.27 | 35.35 | >120.0 |
| Region of Baiyv Mountains | | Yangjiaqu Guojialing at Jingbian County | 108.91 | 37.51 | 194.2 |
|  |  | Ximawan Taoshuqu at Jingbian County | 108.50 | 37.29 | >151.0 |
|  |  | Diqingyuan at Hengshan County | 109.22 | 37.64 | 197.0 |
|  |  | Xiluhe River in Zhenjing County | 108.83 | 37.47 | ~100.0 |
| Weihe Valley in Shaanxi Province | Northern Weihe Plateau | Baishui County | 106.91 | 35.40 | ~70.5 |
|  |  | Wang Village in Heyang County | 110.34 | 35.15 | ~80.0 |
|  |  | Hanjing in Pucheng City | 109.60 | 35.12 | ~90.0 |
|  |  | Emao Village in Heyang County | 110.18 | 35.30 | 73.5 |
|  | Region of Weihe Valley | Sigou Jiaojiawan in Baoji City | 106.98 | 34.44 | ~170.0 |
|  |  | Liujiagou in Qianyang County | 106.98 | 34.60 | ~115.0 |
|  |  | Changshoushan at Baoji City | 107.13 | 34.38 | >87.0 |
|  |  | Qijiayuan at Mei County | 107.79 | 34.30 | ~90.0 |
|  |  | Shuijiaju at Lantian County | 109.37 | 34.24 | 125.7 |
|  |  | You River at Weinan City | 109.55 | 34.37 | 77.5 |
|  |  | W7 hole at Yan Village in Weinan City | 109.59 | 34.44 | 128.0 |
|  |  | Yangjiashanyuan in Baoji City | 106.60 | 34.52 | 73.9 |
|  | Northern piedmont of Qinling Mountains | Lijiaping at Lantian County | 109.57 | 34.12 | ~60.0 |
|  |  | Nanmigou – Chenjiawozi | 109.59 | 33.91 | 87.5 |
| Region of Shanxi Province | | Donggou at Jia Village in Huo County | 111.77 | 36.57 | 50.4 |
|  |  | Chaizhuang Village in Xiangfen County | 111.43 | 35.81 | >65.9 |
|  |  | Dingo Village in Xiangfen County | 111.43 | 35.85 | 21.7 |
|  |  | Jingsheng Village in Lingshi County | 111.88 | 36.90 | ~25.0 |
|  |  | Yujiazhai Village in Yangyuan County | 113.99 | 40.12 | ~27.0 |
| East of Taihang Mountains | | Linchi Dongtai Village in Zouping County | 117.81 | 36.75 | ~10.0 |
|  |  | Linge Village in Penglai City | 120.70 | 37.80 | ~8.0 |
|  |  | Houkou at Changshantuoji Island | 120.76 | 38.17 | <15.0 |
|  |  | Dongzhaitang Village in Xi Mountain of Beijing City | 115.71 | 39.99 | ~13.0 |


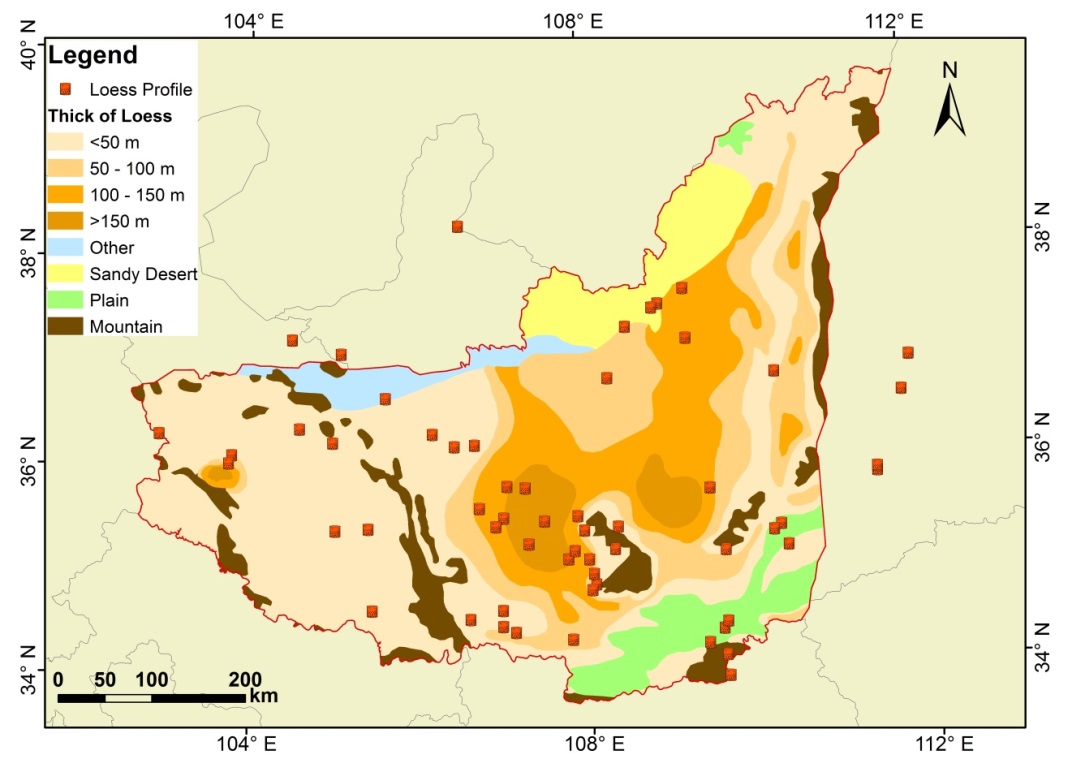


**Figure S14. Location of typical loess profile in Loess Plateau (with scope of NA)**

During estimating the loess volume of the Chinese Loess Plateau, the loess thickness were divided into four classifications (Fig. S13): <50 m, 50~100 m, 100~150 m, and >150 m ^45^. Therefore, during calculation processes, the mean, maximum, and the minimum values were employed for further data processing (Table S6). In addition, the bulk density of the loess is spatially heterogeneous ^46, 48-50^ (Table S7). Therefore, here we use the mean bulk density (1,500 kg m^-3^) for further data processing, and the loess volume estimations of the Chinese Loess Plateau are shown in Table S8.

**Table S6. Mean, maximum, and the minimum values of loess thickness**

| Thickness classification (m) | Mean (m) | Maximum (m) | Minimum (m) |
| --- | --- | --- | --- |
| < 50 | 25 | 50 | 10 |
| 50 - 100 | 75 | 100 | 50 |
| 100 - 150 | 125 | 150 | 100 |
| > 150 | 175 | 200 | 150 |

**Table S7. Bulk density of loess in different regions**

| Location | Loess bulk density  (kg m^-3^) | Paleosol bulk density  (kg m^-3^) | Loess-paleosol  (kg m^-3^) | References |
| --- | --- | --- | --- | --- |
| Luochuan | 1,250 – 1,720 (1,470) | 1,290 - 1,860 (1,630) | 1,280 - 1,860 | 48 |
| Baicaoyuan | — | — | 1,050 - 1,250 |  |
| Lingtai | 1,850 - 1,980 | 2,050 - 2,180 | 1,850 - 2,180 | 49 |

**Note: The mean values are shown in parentheses**

**Table S8. Loess volume estimation in the Chinese Loess Plateau**

| Thickness classification (m) | | | Area (×10^10^ m^2^) | | Volume (×10^9^ m^3^) | | Bulk density (kg m^-3^) | quantity (×10^11^ kg) | |
| --- | --- | --- | --- | --- | --- | --- | --- | --- | --- |
|  |  |  | MA | NA | MA | NA |  | MA | NA |
| <50 | M  e  a  n | 25 | 22.46 | 11.28 | 5,615.59 | 2,820.32 | 1,500 | 84,233.92 | 42,304.81 |
| 50-100 |  | 75 | 4.78 | 4.52 | 3,560.65 | 3,391.70 |  | 53,409.68 | 50,875.45 |
| 100-150 |  | 125 | 3.96 | 3.95 | 4,957.32 | 4,932.95 |  | 74,359.84 | 73,994.21 |
| >150 |  | 175 | 1.05 | 1.05 | 1,835.14 | 1,835.14 |  | 27,527.12 | 27,527.12 |
| Total |  | — | 32.22 | 20.80 | 15,968.70 | **12,980.11** |  | 239,530.56 | **194,701.59** |
| <50 | M  a  x  i  m  u  m | 50 | 22.46 | 11.28 | 11,231.19 | 5,640.64 | 1,500 | 168,467.85 | 84,609.62 |
| 50-100 |  | 100 | 4.78 | 4.52 | 4,747.53 | 4,522.26 |  | 71,212.90 | 67,833.93 |
| 100-150 |  | 150 | 3.96 | 3.95 | 5,948.79 | 5,919.54 |  | 89,231.81 | 88,793.06 |
| >150 |  | 200 | 1.05 | 1.05 | 2,097.30 | 2,097.30 |  | 31,459.57 | 31,459.57 |
| Total |  | — | 32.22 | 20.80 | 24,024.81 | **18,179.74** |  | 360,372.13 | **272,696.17** |
| <50 | M  i  n  i  m  u  m | 10 | 22.46 | 11.28 | 2,246.24 | 1,128.13 | 1,500 | 33,693.57 | 16,921.92 |
| 50-100 |  | 50 | 4.78 | 4.52 | 2,373.76 | 2,261.13 |  | 35,606.45 | 33,916.97 |
| 100-150 |  | 100 | 3.96 | 3.95 | 3,965.86 | 3,946.36 |  | 59,487.87 | 59,195.37 |
| >150 |  | 150 | 1.05 | 1.05 | 1,572.98 | 1,572.98 |  | 23,594.67 | 23,594.67 |
| Total |  | — | 32.22 | 20.80 | 10,158.84 | **8,908.59** |  | 152,382.57 | **133,628.93** |

**Note: MA refers to the area of CLP defined as 47.8×10^4^ km^2^, and NA refers to that is defined as 26.91×10^4^ km^2^.**

# S11 Age of the Badain Jaran Desert

At present, there are still disputes on the age of the Badain Jaran Desert, which varied from 11.7 ka to 2,580 ka. The age of the Desert acquired by previous studies are shown in Table S9.

**Table S9. Age of the Badain Jaran Desert**

| **Formation Era** | **Age (ka BP)** | **References** |
| --- | --- | --- |
| Q3 3-Q1 4 | ^a^ ~11.7 | 51(孙培善, et al., 1964) |
| Q_1_ | ~781 | 52 |
| N_2_-Q_1_ | ~2,580 | 53 |
| Q_1_ | ~2,500 | 54 |
| LGM | 18.5±1.5 | 55 |
| Q_1_ | ~2,000 | 56 |
| Q_1_ | 1,200 – 1,100 | 43 |

***Note*: Both ‘a’ and “~” represents the estimated age.**

# S12 Erosion rates in the Chinese Loess Plateau

The erosion rates of the Chinese Loess Plateau suggested by previous studies are shown in Table S10. Following data in Table S10, the erosion rates of Chinese Loess Plateau during the past 25 ka are summarized in Table S11.

**Table S10. Erosion rates of the Chinese Loess Plateau**

| Period | | Erosion rate  (×10^9^ t a^-1^) | Transports of Yellow River  (×10^9^ t a^-1^) | Deposits in Lower Yellow River/ Yellow River Delta/ Bohai Sea and Huanghai Sea (×10^9^ t a^-1^) | References |
| --- | --- | --- | --- | --- | --- |
| 1951 – 1979AD | | 1.21 | 1.34 | — | 57 |
| 1980 – 1999AD | | 0.66 | 0.73 |  |  |
| 2000 – 2010AD | | 0.29 | 0.32 |  |  |
| 1976 – 1986AD | | — | — | Deposition depth ~15 m | 58 |
| Before 1988a | | 2.45 | 1.60 | Deposition in lower reaches 10 cm a^-1^ | 59 |
| Early Holocene | | 0.22 | 0.24 |  | 60 |
| Middle Holocene | | 0.96 | 1.07 |  |  |
| Late Holocene | 1020BC-1194AD | 1.04 | 1.16 |  |  |
|  | 1494-1855AD | 1.20 | 1.33 |  |  |
|  | 1919-1949AD | 1.51 | 1.68 |  |  |
|  | 1494-1980AD | 1.47 | 1.63 |  |  |
| 1950-1977AD | | 1.8 | 1.80 | — | 61 |
| 1950-1960AD | | 1.53 | 1.70 | — | 62 |
| 1933-1958AD | | 1.57 | 1.75 |  |  |
| Before 2300 a BP | | 0.80 | 1.00 | 1.00 | 63 |
| 340BC-200BC | | 0.36 | 0.40 | 0.40 |  |
| 200BC-60AD | | 0.90 | 1.00 | 1.00 |  |
| 50AD-600AD | | 0.54 | 0.60 | 0.60 |  |
| 600AD-present | | 1.08 | 1.20 | 1.20 |  |
| 1956-1988AD | | 1400-5700 t km^-2^ a^-1^ | — | — | 64 |
|  |  | 3270-6030 t km^-2^ a^-1^ |  |  |  |
|  |  | >8000 t km^-2^ a^-1^ |  |  |  |
| 1973-1977AD | | 4500-15851 t km^-2^ a^-1^ | — | — | 65 |
| 1000 a BP | | 0.24-0.36 | 0.27-0.40 | — | 66 |
| 1955-1989AD | | 1.54 | — | — | 67 |
| 1919-1958AD | | 1.44 | 1.60 | — | 68 |
| 150-75 ka BP | | 0.045 | 0.05 | — | 69 |
| 75-10 ka BP | | 0.023 | 0.025 |  |  |
| 10-4 ka BP | | 0.045 | 0.05 |  |  |
| 4000BC-225BC | | 0.09 | 0.1 |  |  |
| 225BC-210AD | | 0.18 | 0.2 |  |  |
| 210-600AD | | 0.09 | 0.1 |  |  |
| 600-740AD | | 0.18 | 0.2 |  |  |
| 740-960AD | | 0.18 | 0.2 |  |  |
| 960-1370AD | | 0.54 | 0.6 |  |  |
| 1370-1800AD | | 0.09 | 1 |  |  |
| 1800-1957AD | | 1.44 | 1.6 |  |  |
| 1958-1980AD | | 1.26 | 1.4 |  |  |
| 1981-2000AD | | 0.45 | 0.5 |  |  |
| MISI(12 ka BP-present) | | 0.030 | — | — | 70, 71 |
| MIS2-4(74 ka BP-12 ka BP) | | 0.015 |  |  |  |
| MIS5(130 ka BP-74 ka BP) | | 0.013 |  |  |  |
| MIS6(190 ka BP-130 ka BP) | | 0.024 |  |  |  |
| MIS7(250 ka BP-190 ka BP) | | 0.014 |  |  |  |
| 1128-1855AD | | 0.90 | 1.0 | — | 72 |
| 1955-1989AD | | 1.61 | — | — | 73 |
| Before 2000 a BP | | 0.45 | 0.50 | — | 74 |
| 2000 a BP-1000 a BP | | 0.90-1.08 | 1.00-1.20 |  |  |
| 1000 a BP-present | | 1.08-1.17 | 1.20-1.30 |  |  |
| Before 2300 a BP | | 0.97 | 1.08 | — | 75 |
| 1963-2011AD | | 1.59 | — | — | 76 |

**Note:** From 1855 to 1968 A.D., the deposition ratios in Lower Yellow River: Alluvial fan : Delta : Deep Sea = 64% : 33% : 4% ^77^. The erosion rate of Chinese Loess Plateau was 90% × Tansports of the Yellow River ^60, 72, 74^. The area of Chinese Loess Plateau was taken as 32.23×10^4^ km^2 70^.

**Table S11. The erosion rates during the past 25 ka in the Chinese Loess Plateau**

| Period | Erosion rates  (km^3^ a^-1^) | Cumulative amount  (km^3^) |
| --- | --- | --- |
| 250-190 ka BP | 0.01 | 600.00 |
| 190-150 ka BP | 0.02 | 800.00 |
| 150-75 ka BP | 0.03 | 2250.00 |
| 75-10 ka BP | 0.02 | 1300.00 |
| 10-7 ka BP | 0.1 | 300.00 |
| 7-4 ka BP | 0.35 | 1050.00 |
| 2300BC-200BC | 0.57 | 1197.00 |
| 200BC-210AD | 0.52 | 213.20 |
| 210-600AD | 0.36 | 140.40 |
| 600-740AD | 0.12 | 16.80 |
| 740-960AD | 0.12 | 26.40 |
| 960-1370AD | 0.44 | 180.40 |
| 1370-1855AD | 0.78 | 378.30 |
| 1855-1949AD | 0.98 | 92.12 |
| 1949-1979AD | 1.01 | 30.30 |
| 1980-1999AD | 0.44 | 8.36 |
| 2000-2010AD | 0.19 | 1.90 |
| Sum | **0.034** (weighted mean) | **8585.18** |

**Note:** The bulk density of loess is 1,500 kg m^-3^ ^75^.

# References

1 Cable, M. & French, F. *The Gobi desert*. (London: Hodder and Stoughton, 1943).

2 Cooke, R. U. Stone pavement in deserts. *Ann. Assoc. Am. Geogr.* **60**, 560-577 (1970).

3 Wang, X., Xia, D., Wang, T., Xue, X. & Li, J. Dust sources in arid and semiarid China and southern Mongolia: Impacts of geomorphological setting and surface materials. *Geomorphology* **97**, 583-600, 10.1016/j.geomorph.2007.09.006 (2008).

4 Bryant, R. G. Monitoring hydrological controls on dust emissions_preliminary observations from Etosha Pan, Namibia. *The Geographical Journal* **169**, 131-141 (2003).

5 Pullen, A. *et al.* Qaidam Basin and northern Tibetan Plateau as dust sources for the Chinese Loess Plateau and paleoclimatic implications. *Geology* **39**, 1031-1034, 10.1130/g32296.1 (2011).

6 Bryant, R. G., Bigg, G. R., Mahowald, N. M., Eckardt, F. D. & Ross, S. G. Dust emission response to climate in southern Africa. *Journal of Geophysical Research* **112**, 10.1029/2005jd007025 (2007).

7 Zhang, K., Qu, J., Zu, R. & Ta, W. Research on the Characteristics of Sand-Driving Wind Over Gobi/Mobile Sand Surface. *Environmental Geology* **54**, 411-416 (2008).

8 Cooke, R. U. & Smalley, I. J. Salt weathering in deserts. *Nature* **220**, 1226-1227 (1968).

9 Goudie, A. S. Experimental salt weathering of limestones in relation to rock properties. *Earth Surface Processes and Landforms* **24**, 715-724 (1999).

10 McFadden, L. D., Eppes, M. C., Gillespie, A. R. & Hallet, B. S. Physical weathering in arid landscapes due to diurnal variation in the direction of solar heating. *Bulletin Geological Society of America* **117**, 161-173 (2005).

11 Viles, H. A. Microclimate And weathering in the central Namib desert. *Geomorphology* **67**, 189-209 (2005).

12 Viles, H. A. & Goudie, A. S. Rapid salt weathering in the coastal Namib desert: Implications for landscape development. *Geomorphology* **85**, 49-62, 10.1016/j.geomorph.2006.03.025 (2007).

13 Liu, Z. *et al.* Climatic and tectonic controls on weathering in south China and Indochina Peninsula: Clay mineralogical and geochemical investigations from the Pearl, Red, and Mekong drainage basins. *Geochemistry Geophysics Geosystems* **8**, Q05005, 10.1029/2006GC001490 (2007).

14 Dong, Z., Liu, X., Wang, H. & Wang, X. Aeolian sand transport: a wind tunnel model. *Sedimentary Geology* **161**, 71-83, 10.1016/S0037-0738(02)00396-2 (2003).

15 Wang, X., Dong, Z., Yan, P., Yang, Z. & Hu, Z. Surface sample collection and dust source analysis in northwestern China. *Catena* **59**, 35-53, 10.1016/j.catena.2004.05.009 (2005).

16 Wang, X., Hasi, E., Zhou, Z. & Liu, X. Significance of variations in the wind energy environment over the past 50 years with respect to dune activity and desertification in arid and semiarid northern China. *Geomorphology* **86**, 252-266, 10.1016/j.geomorph.2006.09.003 (2007).

17 Wang, X., Zhou, Z., Dong, Z. & Chen, F. Do humans create mineral dust in northwest China? *Environmental Geology* **48**, 609-614, 10.1007/s00254-005-1317-z (2005).

18 Wang, X. *et al.* Characteristics of the Gobi desert and their significance for dust emissions in the Ala Shan Plateau (Central Asia): An experimental study. *Journal of Arid Environments* **81**, 35-46, 10.1016/j.jaridenv.2012.01.014 (2012).

19 Eshel, G., Levy, G. J., Mingelgrin, U. & Singer, M. J. Critical evaluation of the use of laser diffraction for particle-size distribution analysis. *Soil Science Society of America Journal* **68**, 736-743 (2004).

20 Cheng, H. *et al.* Aeolian creeping mass of different grain sizes over sand beds of varying length. *Journal of Geophysical Research: Earth Surface* **120**, 1404-1417, 10.1002/2014jf003367 (2015).

21 Cheng, H. *et al.* Experimental evidence of larger-than-predicted aeolian flux, (in preparation). (2016).

22 Bagnold, R. A. *The Physics of Wind Blown Sand and Desert Dunes*. 265 pp. (New York: William Morrow, 1941).

23 Sherman, D. J. *et al.* Recalibrating aeolian sand transport models. *Earth Surface Processes and Landforms* **38**, 169-178, 10.1002/esp.3310 (2013).

24 Zou, X. *et al.* Spatial variation of topsoil features in soil wind erosion areas of northern China. *Catena* **167**, 429-439, 10.1016/j.catena.2018.05.022 (2018).

25 Andreotti, B. A two-species model of aeolian sand transport. *Journal of Fluid Mechanics* **510**, 47-70, 10.1017/s0022112004009073 (2004).

26 Elbelrhiti, H., Claudin, P. & Andreotti, B. Field evidence for surface-wave-induced instability of sand dunes. *Nature* **437**, 720-723, 10.1038/nature04058 (2005).

27 Hersen, P., Douady, S. & Andreotti, B. Relevant length scale of barchan dunes. *Phys Rev Lett* **89**, 264301, 10.1103/PhysRevLett.89.264301 (2002).

28 Pahtz, T., Kok, J. F., Parteli, E. J. & Herrmann, H. J. Flux saturation length of sediment transport. *Phys Rev Lett* **111**, 218002, 10.1103/PhysRevLett.111.218002 (2013).

29 Fryrear, D. W. & Saleh, A. Wind Erosion: Field Length. *Soil Science* **161**, 398-404 (1996).

30 Gillette, D. A., Herbert, G., Stockton, P. H. & Owen, P. R. Causes of the fetch effect in wind erosion. *Earth Surface Processes and Landforms* **21**, 641-659 (1996).

31 Li, Z. & Zhang, Q. Evolution of streamwise sand transport with distance. *Journal of Desert Research* **26**, 189-193 (2006).

32 Shao, Y. & Raupach, M. R. The overshoot and equilibration of saltation. *Journal of Geophysical Research Atmospheres* **97**, 20559-20564 (1992).

33 Lettau, K. & Lettau, H. *In Exploring the World’s Driest Climate*. 110-147. (University of Wisconsin-Madison: Center for Climatic Research, 1978).

34 Sauermann, G., Kroy, K. & Herrmann, H. J. Continuum saltation model for sand dunes. *Physical Review E Statistical Nonlinear & Soft Matter Physics* **64**, 031305 (2001).

35 Nalpanis, P., Hunt, J. C. R. & Barrett, C. F. Saltating particles over flat beds. *Journal of Fluid Mechanics* **251**, 661-685 (1993).

36 Wang, X., Zhou, Z. & Dong, Z. Control of dust emissions by geomorphic conditions, wind environments and land use in northern China: An examination based on dust storm frequency from 1960 to 2003. *Geomorphology* **81**, 292-308, 10.1016/j.geomorph.2006.04.015 (2006).

37 Wang, X., Hua, T., Lang, L. & Ma, W. Spatial differences of aeolian desertification responses to climate in arid Asia. *Global and Planetary Change* **148**, 22-28, 10.1016/j.gloplacha.2016.11.008 (2017).

38 Zhang, Q., Wang, S. & Wei, G. A study on parameterization of local land-surface physical processes on the Gobi of Northwest China. *Chinese Journal of Geophysics-Chinese Edition* **46**, 616-623 (2003).

39 Chen, S. *et al.* Comparison of dust emissions, transport, and deposition between the Taklimakan Desert and Gobi Desert from 2007 to 2011. *Science China Earth Sciences* **60**, 1338-1355, 10.1007/s11430-016-9051-0 (2017).

40 Zhu, Z. & Cui, S. The problem of desertification in south China. *Journal of Desert Research* **16**, 331-337 (1996).

41 Zhu, J., Wang, N., Chen, H., Dong, C. & Zhang, H. Study on boundary and the area of Badain Jaran Desert based on remote sensing imagery. *Progress in Geography* **29**, 1087-1094 (2010).

42 Dong, Z., Wang, T. & Wang, X. Geomorphology of the megadunes in the Badain Jaran Desert. *Geomorphology* **60**, 191-203, 10.1016/j.geomorph.2003.07.023 (2004).

43 Wang, F. *et al.* Formation and evolution of the Badain Jaran Desert, North China, as revealed by a drill core from the desert centre and by geological survey. *Palaeogeography, Palaeoclimatology, Palaeoecology* **426**, 139-158, 10.1016/j.palaeo.2015.03.011 (2015).

44 Wang, Y. & Sadao, S. *The New Development of Loess Studies in China*. (Xi'an: People's Publishing House, 1985).

45 Zhang, T. *Contention of Key Issues in China's Loess Plateau*. (Beijing: China Environmental Science Press, 1993).

46 Liu, T. *Loess deposition in China*. (Beijing: Science Press, 1965).

47 Zhang, Z., Zhang, Z. & Wang, Y. *Loess in China*. (Beijing: Geological Publishing House, 1989).

48 Jin, C., Zhang, L., Han, J. & Liu, D. Characteristics of volumetric weight in loess-paleosols since the last interglacial. *Journal of Jilin University (Earth Science Edition)* **38**, 801-805, 10.13278/j.cnki (2008).

49 Sun, Y., An, Z., Zhou, J. & Lu, X. Dry bulk density of loess samples measured by the oil-soaked method. *Geological Review*, 220-224. (2000).

50 Zhao, J. *Illuvial Theory and Environmental Evolution of Loess Plateau*. 61-67. (Beijing: Science Press, 2002).

51 Sun, P. & Sun, D. *Primary study on hydrogeology of western Inner Mongolian Plateau - Research of Sand Control, No.6*. [Sun Peishan, Sun Deqin. 1964. Primary study on hydrogeology of western Inner Mongolian Plateau - Research of Sand Control, No.6 [M]. Beijing: Science Press.] edn, (Beijing: Science Press, 1964).

52 Tan, J. *The local type of Alaxa Desert in Inner Mongolia - Geographical Collected Papers, No.8*. (Beijing: Science Press, 1964).

53 Wang, Z. *Reports on Investigation of Badain Jaran Desert*. 1959).

54 Gao, Q., Dong, G., Li, B. & Zou, X. Evolution of southern fringe of Badain Jaran Desert since late Pleistocene. *Journal of Desert Research* **15**, 345-352 (1995).

55 Yang, X. Landscape evolution and paleoclimate in the deserts of northwestern China, with a special reference to Badain Jaran and taklamakan. *Chinese Science Bulletin* **46**, 6-11 (2001).

56 Yan, M., Wang, G., Li, B. & Dong, G. Formation and Growth of High Megadunes in Badain Jaran Desert. *Acta Geologica Sinica* **56**, 83-91 (2001).

57 Wang, S. *et al.* Reduced sediment transport in the Yellow River due to anthropogenic changes. *Nature Geoscience* **9**, 38-41, 10.1038/ngeo2602 (2015).

58 Bornhold, B. D. *et al.* Sedimentary framework of the modern Huanghe (Yellow River) delta. *Geo-Marine Letters*, 77-83 (1986).

59 Fu, B. Soil erosion and its control in the loess plateau of China. *Soil Use and Management* **5**, 76-82 (1989).

60 Jing, K. & Chen, Y. Preliminary study of the erosion environment and rates on the Loess Plateau. *Geographical Research* **2**, 1-11 (1983).

61 Zhu, X. Proper development and carefull protection of land resources in the Loess Plateau. *Scientia Geographica Sinica* **4**, 97-105 (1984).

62 Ren, M. & Shi, Y. Sediment discharge of the Yellow River and its effect on sedimentation of the Bohai and Yellow Sea. *Scientia Geographica Sinica* **6**, 1-13 (1986).

63 John, D. M., Qin, Y., Ren, M. & Yoshiki, S. Man's Influence on the Erosion and Transport of Sediment by Asian Rivers: The Yellow River (Huanghe) Example. *The Journal of Geology* **95**, 751-762, 10.1086/629175 (1987).

64 Zhang, X., Higgitt, D. L. & Walling, D. E. A preliminary assessment of the potential to use ^137^Cs to estimate the rates of soil erosion in Loess Plateau of China. *Geochemica*, 212-218 (1991).

65 Zhang, X., Walling, D. E., Quine, T. A. & Wen, A. Use of reservoir deposits and caesium-137 measurement to investigate the erosional response of a small drainage basin in the rolling Loess Plateau region of China. *Land Degradation & Development* **8**, 1-16 (1997).

66 Yoshiki, S., Yang, Z. & Kazuaki, H. The Huanghe (Yellow River) and Changjiang (Yangtze River) deltas_ a review on their characteristics, evolution and sediment discharge during the Holocene. *Geomorphology* **41**, 219-231 (2001).

67 Wang, W. & Jiao, J. Temporal and spatial variation features of sediment yield intensity on Loess Plateau. *Acta Geologica Sinica* **57**, 210-217 (2002).

68 Ren, M. The past, present and future of sediment discharge of the Yellow River, China. *Science (in Chinese)* **55**, 37-40 (2003).

69 Ren, M. Sediment discharge of the Yellow River, China, past, present and future – a synthesis. *Advances in Earth Science* **21**, 551-563 (2006).

70 Li, L. & Lu, H. A preliminary quantitative estimation of the sedimentation and erosion rates of loess deposits in Chinese Loess Plateau over the past 250 ka. *Acta Geologica Sinica* **65**, 37-52 (2010).

71 Zhang, X. Y., Lu, H. Y., Richard, A. & Gong, S. L. Atmospheric dust loadings and their relationship to rapid oscillations of the Asian winter monsoon climate: two 250-kyr loess records. *Earth and Planetary Science Letters* **202**, 637-643 (2002).

72 Wang, H. *et al.* Recent changes of sediment flux to the western Pacific Ocean from major rivers in East and Southeast Asia. *Earth-Science Reviews* **108**, 80-100, 10.1016/j.earscirev.2011.06.003 (2011).

73 Liu, Y., Fu, B., Lü, Y., Wang, Z. & Gao, G. Hydrological responses and soil erosion potential of abandoned cropland in the Loess Plateau, China. *Geomorphology* **138**, 404-414, 10.1016/j.geomorph.2011.10.009 (2012).

74 Zhao, G., Mu, X., Wen, Z., Wang, F. & Gao, P. Soil Erosion, Conservation, and Eco-Environment Changes in the Loess Plateau of China. *Land Degradation & Development* **24**, 499-510, 10.1002/ldr.2246 (2013).

75 Nie, J. *et al.* Loess Plateau storage of Northeastern Tibetan Plateau-derived Yellow River sediment. *Nat Commun* **6**, 8511, 10.1038/ncomms9511 (2015).

76 Li, M., Yao, W., Shen, Z., Yang, J. & Yang, E. Erosion rates of different land uses and sediment sources in a watershed using the 137Cs tracing method: field studies in the Loess Plateau of China. *Environmental Earth Sciences* **75**, 10.1007/s12665-015-5225-6 (2016).

77 Shi, C. & Zhang, D. A sediment budget of the lower Yellow River, China, over the period from 1855 to 1968. *Geografiska Annaler Series A-Physical Geography* **87A**, 461-471 (2005).
